# Sensory Cues Involved in Social Facilitation of Reproduction in *Blattella germanica* Females

**DOI:** 10.1371/journal.pone.0055678

**Published:** 2013-02-06

**Authors:** Adrienn Uzsák, Coby Schal

**Affiliations:** Department of Entomology and W. M. Keck Center for Behavioral Biology, North Carolina State University, Raleigh, North Carolina, United States of America; University of Osnabrueck, Germany

## Abstract

Cockroaches, like many other animal species, form aggregations in which social stimuli from conspecifics can alter the physiology, morphology, or behavior of individuals. In adult females of the German cockroach, *Blattella germanica*, social isolation slows oocyte development, sexual maturation, and sexual receptivity, whereas social interactions as minimal as between just two females accelerate reproduction; however, the sensory modalities and pathways that mediate these physiological and behavioral changes are poorly understood. We explored the roles of visual, olfactory, and tactile cues in the reproductive physiology of German cockroach females, and whether their effects are species-specific and related to circadian time. Our results show that tactile cues are the primary sensory input associated with social conditions—with no evidence for involvement of the visual and olfactory systems—and that the antennae play an important role in the reception of these tactile cues. This conclusion is supported by the observation that interactions with other insect species of similar or larger size and with similar antennal morphology also stimulate oocyte development in *B. germanica*. Social facilitation of reproduction is expected to be influenced by the circadian timing system, as females engage in more social contact during the day when they shelter in aggregations with conspecifics. Surprisingly, however, the female's reproductive rate was unresponsive to social interactions during the photophase, whereas social interactions as short as two hours during the scotophase were sufficient to induce faster reproduction.

We discuss the adaptive significance of these sensory-neuroendocrine responses in the German cockroach.

## Introduction

Group-living in animals is a critical component of various adaptive behaviors including foraging, food handling, avoiding predators, mate finding and mate choice, and reproduction [1]. In many species, group-living can alter the behavior, morphology, or physiology of individuals, usually promoting survival and greater fitness [1], [2]. This phenomenon—known as “social facilitation” or “grouping effect” [3], [4]—is well described in many insect species, and is a form of group-induced phenotypic plasticity. Thus, grouping can affect larval development [5]–[12], and larval or adult morphology [13]–[18]. In several cockroach species, for example, grouped nymphs develop faster and reach the adult stage sooner than those reared in isolation [7], [8], [12], [19], [20]. The sensory cues that mediate group-induced phenotypic plasticity may derive from direct social interactions or from perceiving the presence of conspecifics without direct contact.

The effects of social interactions on reproduction have been investigated as well, mainly in orthopterans [15], [21]–[25]. For example, when solitarious desert locust adults (*Schistocerca gregaria* (Frosk.) (Orthoptera: Acriidae)) interact socially, a phase change occurs and they produce gregarious-form hatchlings that are larger and darker than those produced by solitarious females [25]. The effects of social conditions on reproduction have also been investigated in two cockroach species, the German cockroach, *Blattella germanica* (L.) (Dictyoptera: Blattellidae) and the brownbanded cockroach, *Supella longipalpa* (Fabricius) (Dictyoptera: Blattellidae). During copulation, physical contact with males and transfer of a spermatophore induce faster oocyte development in females of both species; however, so far the German cockroach appears to be the only cockroach species where females also respond to non-copulatory social interactions, including with other females, with faster sexual maturation and oocyte development [26]–[29]. Juvenile hormone III (JH), a major insect gonadotropic hormone produced and released by the corpora allata (CA), regulates the rate of female reproduction in *B. germanica*, including the synthesis and uptake of vitellogenin and other female-specific proteins, onset of sexual receptivity, production of sexual signals, mating, and the time-course of oviposition [28], [30]–[33]. We recently showed that social interactions with other females can modulate the rate of all JH-dependent events in the female reproductive cycle, as assayed by JH production, oocyte development, and sexual maturation [34].

The sensory pathways through which social interactions modulate the reproductive rate vary widely among animals, but can include various combinations of visual, tactile, chemical (olfactory and/or gustatory) or auditory cues. For example, in some lizards, female ovarian development is stimulated by the sight of displaying males [35]. Similarly, in flamingos, a positive relationship has been revealed between behavioral stimulation from group displays and reproductive success: large flock size on the breeding ground facilitates pair formation and stimulates nesting and breeding [36]. The importance of chemical cues is probably best described in eusocial insects, where pheromones play a central role in the coordination and integration of colony activities such as caste differentiation, division of labor, and foraging [37]. In other insect species, like *S. gregaria*, just a few hours of social interaction are sufficient to induce a behavioral phase-change from solitarious to gregarious individuals. Gregarization is triggered by both the sight and smell of other locusts [38], [39], but direct contact with other locusts is the primary trigger of phase change, and touch-sensitive sensilla on the outer surfaces of the hind femora have been identified as the critical sensory structure for contact stimulation [38]–[41]. In addition, when isolated adult female locusts are placed in a group, tactile stimulation perceived through their antennae causes these females to produce gregarious offspring [25].

In cockroaches, the sensory stimuli responsible for social facilitation of nymphal development have been investigated in *B. germanica* and in the American cockroach, *Periplaneta americana* (L.) (Dictyoptera: Blattidae), but they remain poorly understood. Both species are nocturnal, and in both, visual, olfactory and gustatory cues from conspecifics appear to have little or no effect on nymphal growth, whereas tactile stimulation is sufficient to trigger faster development [7], [8], [12], [42]. The effect of social experience on nymphal development is not species-specific, because nymphs of *B. germanica* grouped with other cockroach species or even locusts grow faster than isolated nymphs [8], [12]. Since different taxa presumably produce different chemical cues, it would appear that chemoreception is less important than tactile stimuli in modulating the developmental rate. This inference is supported by the remarkable observations that contact with a rotating bird feather also stimulated faster development in *B. germanica* nymphs [12]. It is not known which sensory stimuli facilitate faster reproduction in *B. germanica* females.

A proposed model for social facilitation of reproduction in *B. germanica* is that sensory cues associated with social interactions stimulate the central nervous system (CNS), which then accelerates reproduction by lifting inhibition of the CA [26], [34]. However, the sensory modalities and pathways by which such sensory cues are transduced have not been elucidated. Also of interest is whether the effects of the grouping stimuli are modulated by a circadian timing system: Is the “grouping effect” constant or circadian phase-dependent and effective only at specific phases of the photocycle? Social behaviors in cockroaches that are under circadian control include the release of sex pheromones by females [43], [44], behavioral response of males to sex pheromone [45], [46], timing of copulatory behavior [33], [47], and aggressive interactions in males [48]. The German cockroach is night-active, and nymphs and adults aggregate in resting sites during the photophase [49]. We suspected that because females have more opportunities to socially interact with aggregating cockroaches during the photophase, social facilitation of reproduction would be more effective during the photophase than scotophase.

Here, we report on the effects of visual, olfactory, gustatory and tactile stimuli on social facilitation of female reproduction in the German cockroach. Because they are the anterior-most sensory appendage and are involved in social interactions, we hypothesized that antennal contact would be crucial for this “grouping effect”. In addition to chemosensilla, the cockroach antennae house an array of mechanoreceptors [50]. We hypothesized that tactile cues are most important in socially facilitating reproduction, as has been shown for nymph development. We also investigated whether the stimuli responsible for group effects are species-specific in adult females, and whether these effects are linked to a circadian timing system.

## Materials and Methods

### Insects

The *Blattella germanica* colony (American Cyanamid strain, also known as Orlando strain, in lab culture since 1989) was maintained in incubators at 27±1°C, 40–70% relative humidity and under a 12∶12 LD cycle. Cockroaches were allowed continuous access to water and dry rodent diet food pellets (LabDiet 5001 PMI Nutrition International, Brentwood, MO, USA). Newly emerged virgin adult females were separated immediately after eclosion (day 0). Only females of similar size and degree of sclerotization with intact wings were used for all behavioral assays, which were conducted under the same environmental conditions described for colony rearing. We use “paired” to denote pair-housed females, i.e., a *B. germanica* female housed in the same dish with either a conspecific female or another insect, as described below.

### Oocyte dissection and measurements

In *B. germanica*, a single basal (vitellogenic) oocyte matures synchronously in each of approximately 40 ovarioles during the preoviposition period, and the length of the basal oocytes is a reliable measure of the female's reproductive stage because it is highly correlated to JH biosynthesis and JH titer [51], [52]. The ovaries of cold-anesthetized females were dissected under cockroach saline [53] and 10 basal oocytes were randomly measured with the aid of an ocular micrometer in the eyepiece of a dissecting microscope. The lengths of 10 oocytes were averaged for each female and represented a single replicate.

### Diel periodicity of the reproductive response to social interactions

To investigate the effects of transient pairing in otherwise socially isolated females on oocyte maturation, cohorts of newly-eclosed females (day-0) were reared in isolation, but paired daily in small plastic cages (95×95 mm, 90 mm high, Althor Products, Windsor Locks, CT, USA) for various periods (1, 2, and 6 h) either during the middle of their scotophase or photophase. These females were dissected and their oocytes were measured on day 6. Sample size was 19–22 females per treatment.

### Effects of visual cues on oocyte maturation

Newly-eclosed females were either socially isolated or paired with a conspecific female in a plastic Petri dish (90 mm diameter, 15 mm high, Fisher Scientific, Pittsburgh, PA, USA) provisioned with food and water. Some isolated females were allowed an unobstructed view of other females through the tops and bottoms of the plastic dishes, whereas opaque dividers between and around the dishes prevented other isolated females from the same cohort from seeing each other. The eyes of another set of females were painted with opaque nail polish to mask their vision and these females were paired with an intact conspecific female in a Petri dish; newly-eclosed intact control females were either isolated or paired. On day 6, females were dissected and their basal oocytes were measured. Sample size was 18–23 females per treatment.

### Effects of chemical cues on oocyte maturation

Individual newly-eclosed females were socially isolated in Petri dishes. Chemical cues consisted of either cockroach-conditioned filter papers or live cockroaches separated from test subjects by a fine-mesh screen that prevented physical contact. Conditioned filter papers were obtained by placing clean filter papers (Whatman#1, 90 mm diameter) for 7 days in dishes with either (1) 10 German cockroach nymphs or (2) 10 German cockroach females. Volatile chemical cues were provided for 6 days by (1) using fine-mesh mosquito screen between Petri dishes housing isolated females, and (2) placing a screen between a socially isolated female and a group of 15 conspecific females; this design prevented contact between cockroaches. The length of the basal oocytes was measured on day 6, and sample size was 14–20 females per treatment.

### Species-specificity of the “grouping effect” in *B. germanica*

To test whether females respond to social interaction with other insect species, newly-eclosed individual females were placed in Petri dishes with food and water provided *ad libitum* and paired for 6 days with one of the following insects: ant worker (*Camponotus pennsylvanicus*), beetle larva (*Tenebrio molitor*), beetle adult (*Tenebrio molitor*), stink bug adult (*Chinavia hilaris*), smokybrown cockroach nymph (*Periplaneta fuliginosa*), adult fly (*Musca domestica*), adult katydid (*Conocephalus strictus*), adult brownbanded cockroach female (*Supella longipalpa*), and adult camel cricket (*Ceuthophilus maculatus*). The basal oocytes of *B. germanica* females were measured on day 6 and sample size was 17–22 females per treatment.

### Effects of tactile cues on oocyte maturation

Newly-eclosed females were socially isolated in Petri dishes with food and water provided *ad libitum* under five experimental conditions. First we tested the importance of motion by placing a freshly freeze-killed conspecific female into the dish; dead females were replaced daily. In subsequent experiments, mechanical stimuli were provided by glass beads (9 smaller, 4 mm diameter beads and 1 larger, 5 mm diameter bead; Fisher Scientific) placed in each dish with one female. The dish was placed on a rotary shaker (The Waver, VWR Scientific, pitch 6°, speed 4, 22 rpm) so that beads slowly contacted the female in a random fashion. Contact with conspecific females was provided by tethering the wings of a live female and either placing the tethered female inside the dish with the test female or introducing only her antennae and a small portion of the head through a small hole in the side of the Petri dish. The latter treatment was repeated with tethered American cockroach females (*P. americana*). Each experiment lasted for 6 days, when *B. germanica* females were dissected and their basal oocytes were measured. Sample size was 19–22 females per treatment.

In order to differentiate chemical cues from contact cues, we also devised a bioassay for evaluating the effects of antennal contact cues alone. *P. americana* females were selected to provide the contact stimulus. A live *P. americana* female was placed into a 15 ml plastic tube, with only the antennae protruding through a small hole in the tube. The head was covered with parafilm so that only the antennae extended through a hole into a 60×15 mm Petri dish, where a newly-eclosed *B. germanica* female was placed. Four additional treatments included (a) *P. americana* females whose antennae were extracted with hexane to eliminate chemical cues; we washed the antennae sequentially in three vials containing hexane for 30 s each, and then allowed the antennae to dry; (b) *P. americana* females whose antennae were extracted, as described above, but we reapplied on each extracted antenna a lipid extract from *P. americana* females that also contained cuticular hydrocarbons (CHC; see below), (c) *P. americana* females whose antennae were carefully ablated with fine scissors just distal to the pedicel, and (d) *P. americana* females whose antennae were similarly ablated and replaced with an artificial microfibett (1 mm diameter, 40 mm long, smooth surface, Spirit River Inc., Roseburg, OR, USA) glued onto each pedicel, supported by a plastic sleeve. *P. americana* females were replaced every other day during the 6-day-long experiment. On day 6, the oocyte length of *B. germanica* females was measured. Sample size was 14–34 females per treatment.

The re-application of cuticular lipids on the antennae (b above) was conducted as follows. Fifteen freshly-killed *P. americana* females were extracted for 5 min in 75 mL of pentane. The pentane was reduced to ∼2 mL under a gentle stream of N_2_ and filtered through silanized glass wool in a Pasteur pipet to remove particulates. The filtrate was then reduced to dryness and redissolved in 1 mL hexane. A 1 µL aliquot of this solution was combined with 0.1 mL of hexane containing 10 µg of *n*-dotriacontane as an internal standard, and the concentration of the CHC in this solution was determined by gas chromatography. The remaining CHC solution (without the internal standard) was then adjusted to 16 µg of total CHC per 1 µL hexane. We applied 80 µg total CHC (5 µL) to each antenna; this amount was determined by gas chromatography to be the average amount of CHC extracted in 2 min from the antennal cuticular surface of *P. americana* females that had been immobilized for 24 h [54].

### Function of the antennae in receiving social cues that stimulate reproduction

The antennae likely are essential for receiving sensory input that ultimately modulates oocyte growth in *B. germanica*. Therefore we conducted bilateral antennectomies to eliminate these inputs. Females were briefly anesthetized with CO_2_, placed on ice and the flagellum of each antenna was cut with fine scissors just distal to the pedicel. Females were immediately put into one of five treatment groups: (1) isolated intact female (negative control); (2) isolated antennectomized female; (3) intact female paired with an antennectomized female; (4) two antennectomized females; and (5) paired intact females (positive control). Females were housed in plastic 60×15 mm Petri dishes provisioned with food and water *ad libitum* and their oocytes were measured on day 6.

A complementary, less traumatic treatment involved separating two intact females with a wide-mesh metal screen (2 mm mesh opening) which permitted some antennal contact but prevented body contact between the two females.

### Statistical analysis

Data were analyzed with one-way ANOVA for multiple comparisons using SAS® 9.1.3 software (SAS Institute Inc. 2002–2003, Cary, North Carolina). We used PROC GLM to test for the effects of social conditions on oocyte length and JH synthesis as dependent variables. PROC GLM was also used to get the residuals from adjusted model and test whether residuals hold the assumption of homogeneous variances within each treatment. Since data were unbalanced, we used LSMEANS and LSD test was used to compare means at the 0.05 significance level. Variation around the mean is represented by the standard error of the mean (SE).

## Results

### Social facilitation of reproduction is more effective in the scotophase

**Figure 1:**
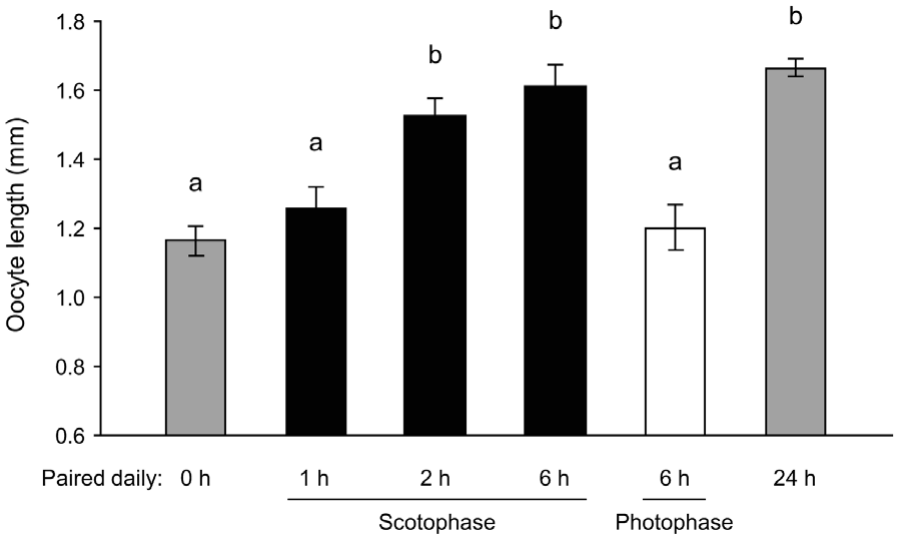
Response of *B. germanica* females to transient social interaction during the photophase and scotophase. Newly-eclosed females were reared either in social isolation (negative control) or paired with a conspecific female (positive control) for the entire 6-day-long experiment. Other females were transiently paired 6 h daily either in the middle of the photophase or the middle of the scotophase. Some females were transiently paired for 1 or 2 h daily in the middle of the scotophase. Mean basal oocyte length ± SE Different letters above the bars indicate significant differences among treatments (ANOVA, LSD, *F*_5, 121_ = 13.69, *P*<0.0001).

Contrary to our expectations, social interactions for 6 h daily only during the photophase did not accelerate the rate of oocyte maturation (oocyte length 1.20±0.06 mm, *N* = 19) relative to social isolation for the entire 6 day period (negative control; 1.19±0.06 mm, *N* = 24) (Fig. 1). In contrast, the presence of another female for the same 6 h period daily, but during the scotophase, significantly enhanced oocyte maturation (1.61±0.06 mm, *N* = 20) to the level of females paired for the entire 6 days (positive controls; 1.66±0.03 mm, *N* = 20) (Fig. 1). A dose (time)-response (oocyte length) experiment revealed that while daily social interaction for just 1 h during the middle of scotophase did not affect oocyte maturation, 2 h of daily social interaction was sufficient to significantly accelerate oocyte maturation in *B. germanica* females (Fig. 1).

### Chemical and visual cues appear to play no role in social facilitation of reproduction

**Figure 2:**
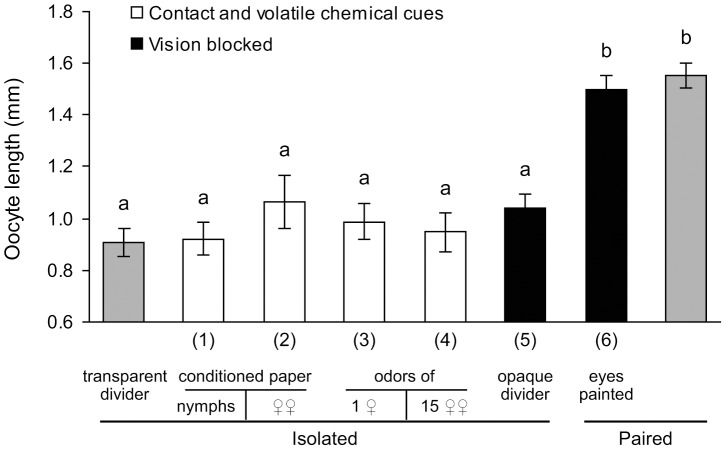
Effects of visual and chemical stimuli on the reproductive rate of *B. germanica* females. Newly-eclosed females were socially isolated in stacked transparent Petri dishes with unobstructed views of other isolated females above and below (isolated; negative control), filter paper contaminated by 10 German cockroach nymphs (1) or 10 conspecific females (2), the odors of one (3), or 15 conspecific females (4) through a fine-mesh screen, or an opaque divider between dishes that prevented them from seeing each other (5). Females paired with another female either had their eyes painted to mask their vision (6), or left untreated (paired; positive control). Variation around the mean is represented by the standard error of the mean (SE). ANOVA: *F*_7, 147_ = 21.72, *P* = 0.0001. Means not sharing a letter are significantly different (LSD, *P*<0.05).

We found no evidence of an effect of either contact or volatile chemical stimuli on the pace of oocyte maturation (Fig. 2). In all treatments involving social isolation and exposure to contact or volatile chemicals, the length of the basal oocytes was not significantly different from the negative controls (females socially isolated during the entire experiment), indicating a lack of response to social interaction via chemical communication. On the other hand, even limited antennal contact through a wide-mesh screen stimulated oocyte maturation relative to solitary females (1.15±0.05 mm, *N* = 18 vs. 0.98±0.05 mm, *N* = 18; *P* = 0.0562), suggesting that antennal contact is pivotal for the grouping effect.

Similarly, visual stimuli also appeared to be unimportant because isolated females exhibited slow oocyte growth whether their view of conspecifics was masked or not. The reproductive rate was the same in isolated females that could see other females and isolated females that were prevented from seeing other females by an opaque divider between their dishes (Fig. 2). Likewise, when females with painted eyes were paired with another female for the 6-day-long experiment, the length of their basal oocytes was approximately the same as in females with intact eyes that were also socially paired (positive controls) (Fig. 2). These results suggest that the suppression of oocyte development in isolated females cannot be overcome by chemical or visual cues from conspecific females or nymphs.

### Effects of social interactions with different insect species on oocyte maturation

**Figure 3:**
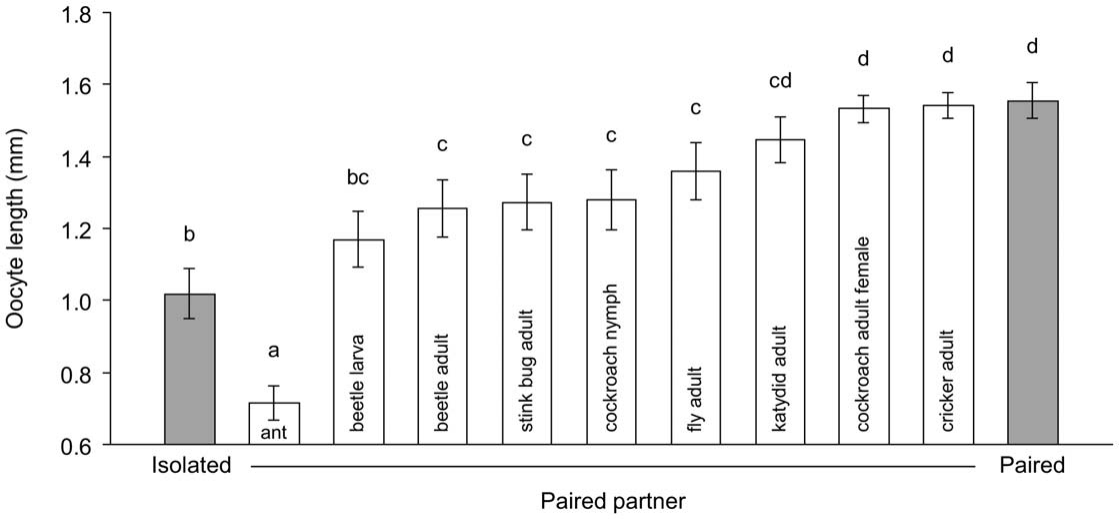
Effects of social interactions with different insect species on the reproductive rate of *B. germanica* females. Females socially isolated during the entire 6-day experiment represent the negative control, and females that were socially paired during the entire experiment represent the positive control. Grey bars represent social pairing for 6 days with different insect species, including a worker ant (*Camponotus pennsylvanicus*), beetle larva (*Tenebrio molitor*), adult beetle (*Tenebrio molitor*), adult stink bug (*Chinavia hilaris*), cockroach nymph (*Periplaneta fuliginosa*), adult fly (*Musca domestica*), adult katydid (*Conocephalus strictus*), adult female cockroach (*Supella longipalpa*), and adult camel cricket (*Ceuthophilus maculatus*). Variation around the mean is represented by the standard error of the mean (SE). ANOVA: *F*_10, 205_ = 15.02, *P* = 0.0001. Means not sharing a letter are significantly different (LSD, *P*<0.05).

Contact with various insect species facilitated oocyte maturation in *B. germanica* females. Social interaction with one heterospecific insect of approximately similar or smaller size than a German cockroach female resulted in moderately faster oocyte maturation than in isolated females (*P*<0.05; Fig. 3). This effect was even more pronounced when females were housed either with a katydid, brownbanded cockroach female, or camel cricket, all of which have longer antennae than *B. germanica* females. Conversely, when females were housed with a beetle larva or with an ant, oocyte maturation was slower (Fig. 3), possibly because of dramatically divergent antennal and body morphology (beetle) or aggressive behavior or defensive secretions (ant).

### Antennal contact mediates social facilitation of reproduction in *B. germanica*

**Figure 4:**
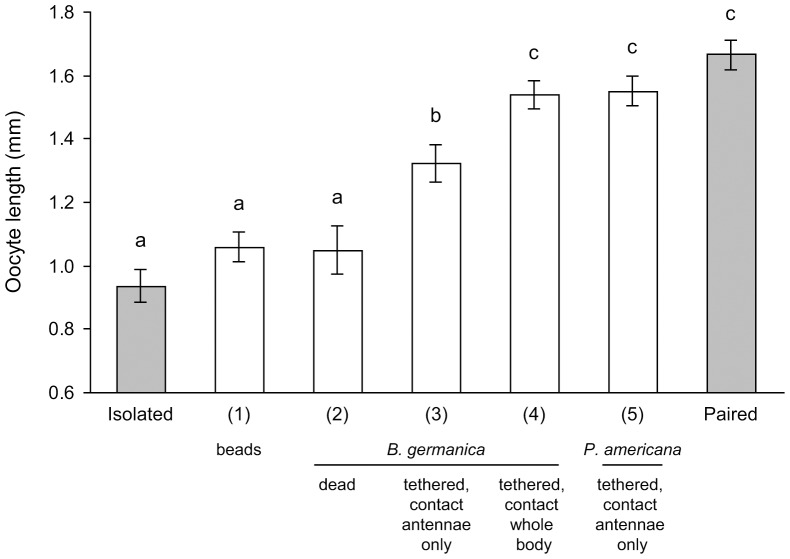
Effects of contact stimuli on the reproductive rate of *B. germanica* females. Newly-eclosed females were either reared in isolation (negative control), allowed to interact with glass beads that were moved to simulate social interaction (1), paired with a freshly-killed conspecific female that was replaced daily (2), allowed to interact with the antennae of a live, conspecific female whose body was tethered either outside the dish (3) or within the dish (4), or allowed to interact with the antennae of an American cockroach female whose body was outside the dish (5). Females paired with a conspecific female for the entire experiment represented the positive control. Mean basal oocyte length ± SE. ANOVA: *F*_6, 136_ = 25.97, *P*<0.0001. Different letters above the bars indicate significant differences among treatments (LSD, *P*<0.05).

The quality of tactile stimuli is important, because intermittent contact with moving glass beads failed to stimulate oocyte maturation in isolated females, whereas contact with moving antennae did (Fig. 4). Moreover, interaction with a freshly-killed conspecific female did not stimulate oocyte maturation, indicating that movement is an important feature of the tactile cue. We found that the degree of social facilitation of reproduction by live conspecific females was related to the amount of their bodies exposed to the test female. Thus, interaction with the antennae and body of a female that was tethered inside the dish stimulated significantly faster oocyte maturation by day 6 (1.54±0.04 mm, *N* = 20) than interaction only with the antennae of a conspecific female whose body was tethered outside the dish (1.32±0.06 mm, *N* = 21) (ANOVA, *P*<0.0001). Notably, however, interaction with the antennae of *P. americana* females (body tethered outside the dish) was as effective as interaction with the whole body of *B. germanica* (1.55±0.04 mm, *N* = 22) (ANOVA, *P*<0.0001) (Fig. 4). These results suggest that both the quality and quantity of social stimuli are important in the social facilitation of reproduction in German cockroach females. The antennae appear to be especially effective in this context, and longer and thicker antennae appeared to be more effective than short and non-filiform antennae.

**Figure 5:**
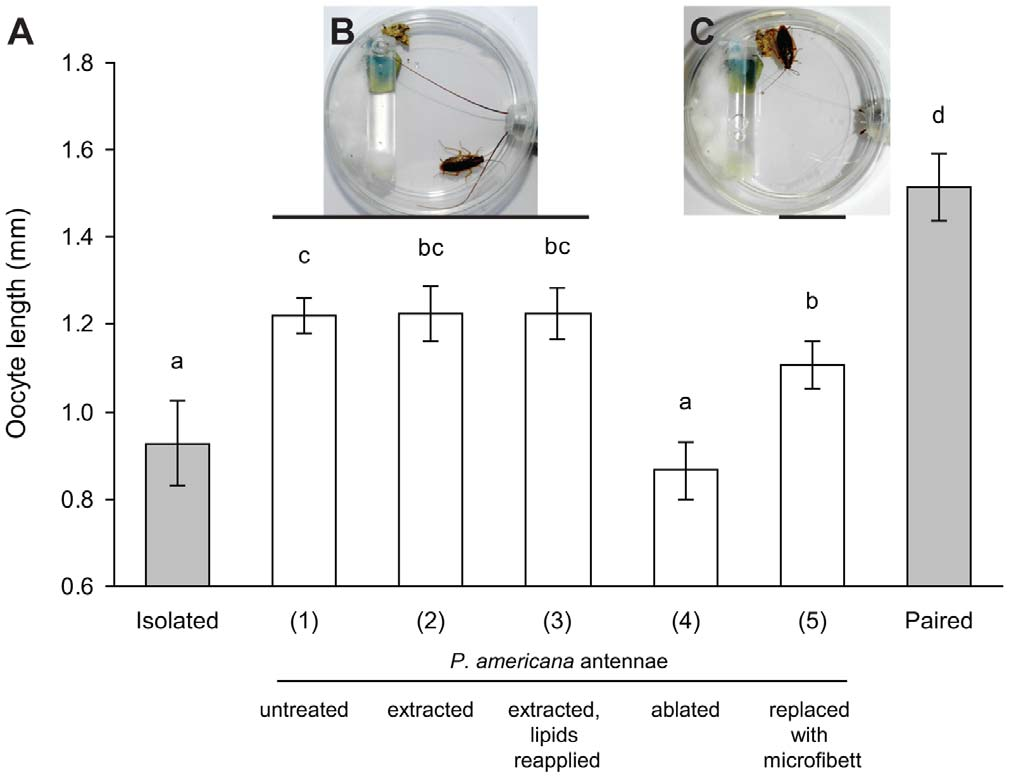
Effects of interactions with the antennae on the reproductive rate of *B. germanica* females. Newly-eclosed females were either socially isolated (negative control), or socially paired for the entire experiment (positive control). An American cockroach female was placed in a plastic tube outside the dish with only its antennae protruding into the dish to interact with the otherwise isolated female. (**A**) Group 1 represents untreated *P. americana* antennae, in group 2 the antennae were extracted with hexane to eliminate chemical cues, and in group 3 the antennae were similarly extracted but a lipid extract from *P. americana* females that also contained cuticular hydrocarbons (CHC) was reapplied on each extracted antenna. In group 4 the *P. americana* antennae were ablated, and in group 5 each ablated antenna was replaced with an artificial microfibett glued onto the pedicel. Mean basal oocyte length ± SE. ANOVA: *F*_5, 137_ = 25.41, *P*<0.0001. Different letters above the bars indicate significant differences among treatments (LSD, *P*<0.05). (**B**) A single *B. germanica* female interacting with the antennae of a female *P. americana* that is restrained within a tube outside the Petri dish. The head of the restrained female is covered with parafilm. (**C**) A single *B. germanica* female interacting with artificial “prosthetic” antennae on a female *P. americana* restrained within a tube outside the Petri dish. The microfibett “antennae” are attached to the antennal scape with a plastic sleeve.

Live antennae present to the test female both mechanosensory and chemical cues. We uncoupled these cues by extracting the antennae of live females with hexane and reapplying a cuticular lipid extract onto the antennae of some females. Females in the control group (group 1; allowed to contact only the antennae of a tethered *P. americana* female; Fig. 5B) matured their oocytes significantly faster than socially isolated females (1.29±0.03 mm, *N* = 34 vs. 0.89±0.05 mm, *N* = 28; respectively) (ANOVA, *P*<0.0001) (Fig. 5A). Similar results were obtained when we removed the cuticular lipids, including CHC, from the *P. americana* antennae (group 2), and also when we reapplied the lipids onto the extracted antennae of *P. americana* females (group 3); in both treatments oocytes grew significantly faster than in the isolated females (1.22±0.06 mm, *N* = 14 and 1.22±0.06 mm, *N* = 14; respectively) (ANOVA, *P*<0.0001) (Fig. 5A). A slightly smaller effect was observed when we ablated the antennae of *P. americana* females and replaced them with artificial (prosthetic) microfibetts (group 4, Fig. 5C; 1.11±0.05 mm, *N* = 19); however, these results were still significantly different from the negative control. Because all our treatments for antennal contact using *P. americana* females showed nearly equivalent results, and because even an artificial material replacing the antenna could induce faster oocyte growth, these results provide compelling evidence that social interactions accelerate the reproductive rate in *B. germanica* females mainly via mechanosensory rather than chemical cues.

### The antennae receive cues that modulate the rate of oocyte maturation in *B. germanica*

**Figure 6:**
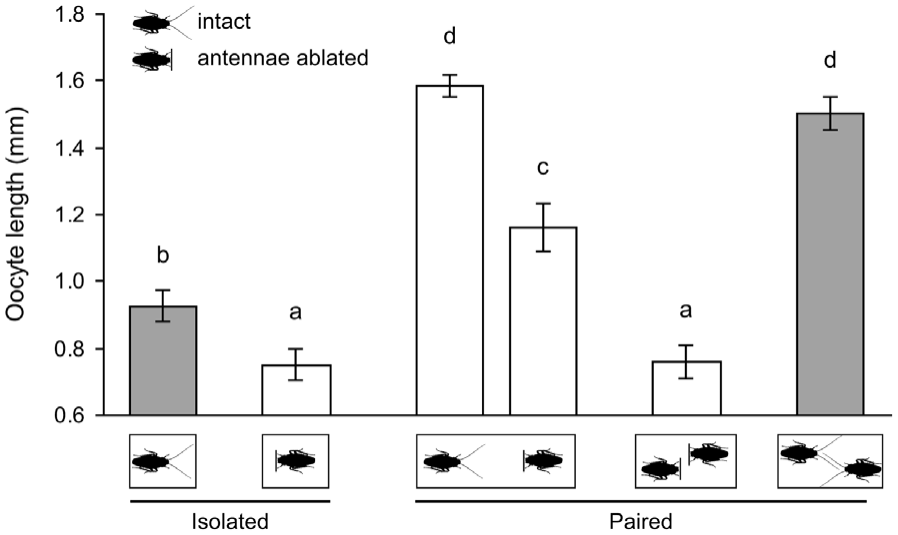
Function of the antennae in receiving social cues that stimulate reproduction. Newly-eclosed females were ice-anesthetized and the flagellum of each antenna was ablated just distal to the pedicel. Socially isolated intact females represent the negative control, and intact females that were socially paired represent the positive control. Other females received one of the following treatments: (1) isolated-housed antennectomized female; (2) an intact female paired with an antennectomized female; and (3) paired antennectomized females. Mean basal oocyte length ± SE. ANOVA: *F*_5, 122_ = 52.29, *P*<0.0001. Different letters above the bars indicate significant differences among treatments (LSD, *P*<0.05).

Bilateral antennectomies of both paired females completely eliminated the “grouping effect” compared to intact paired females (0.76±0.05 mm, *N* = 20 vs. 1.50±0.05 mm, *N* = 26) (ANOVA, *P*<0.0001) (Fig. 6), suggesting that the antennae play a pivotal function in receiving cues from social interactions. Both socially isolated antennectomized females and paired antennectomized females failed to develop their oocytes even to the level of intact isolated females (0.93±0.05 mm, *N* = 20), suggesting that complete ablation of the antennal flagella might have side-effects related to loss of sensory input. Although surgical ablation of the antennae likely eliminated not only the “grouping effect” but other important sensory-based behaviors as well (e.g., feeding, drinking, oriented movement), when an antennectomized female was paired with an intact female the oocytes of the antennectomized female grew significantly (1.16±0.07 mm, *N* = 21), but not as much as in her intact dish-mate (1.58±0.03 mm, *N* = 21). Therefore, the antennae of the intact female appeared to be important for providing social stimuli to the antennectomized female. Nevertheless, these results demonstrate that it is difficult to disentangle the processes of “giving” social stimuli from “receiving” social stimuli because the antennae are used for both.

## Discussion

Paired *B. germanica* females experience robust socially-mediated facilitation of reproduction, an example of phenotypic plasticity, where environmental cues direct development along divergent trajectories, leading to different phenotypes. Our investigations address three major questions regarding the social-facilitation of reproduction in *B. germanica*: (1) What are the major sensory inputs and features of sensory stimuli, and how species-specific are they? (2) How are sensory stimuli received by the female? and (3) What are the sensory-CNS-neuroendocrine transduction pathways that mediate the social facilitation of reproduction? Our results shed light on the influence of socially-derived visual, olfactory, and tactile stimuli on the pace of reproduction in *B. germanica* females. We demonstrated that visual and chemical stimuli are largely ineffective in eliciting faster oocyte growth, but tactile stimulation alone is as effective as housing a female with a conspecific female. The quality of the tactile stimulation is important too, because contact with longer and thicker heterospecific filiform antennae was even more effective than stimulation with conspecific *B. germanica* antennae. Remarkably, modulation of the reproductive rate through social interactions operates only during the scotophase when females are active, and not during the photophase when females are in shelters and in close contact with conspecifics.

### Sensory inputs that facilitate reproduction in *B. germanica*

We designed assays to differentiate among several sensory stimuli from other individuals that might modulate the reproductive rate of female *B. germanica*. Vision was excluded as an important sensory modality in this context because socially isolated females exhibited equally slow oocyte maturation whether they were allowed to see other females or not, and whether their vision was blocked or remained intact. Moreover, in paired females, social interactions equally stimulated oocyte development whether the female's vision was blocked or not. Thus, vision does not appear to be important in the social modulation of the rate of oocyte growth, although it is possible that visual stimuli from conspecifics may play a minor modulatory function in concert with other sensory modalities. This conclusion was not unexpected because *B. germanica* is a nocturnal insect and most behavioral interactions occur in dark places [49].

In contrast to vision, chemical communication is widely used in the German cockroach in many contexts including aggregation [55]–[57], long- and short-range mate attraction, courtship behavior leading to mate choice, and pre- and post-copulatory nuptial exchanges [58]–[62]. It is surprising, therefore, that the strong effects of social interactions on oocyte maturation do not involve chemical stimuli: Neither contact nor volatile chemical cues from conspecifics were sufficient to induce changes in oocyte maturation of isolated females. Because contact stimulation with whole insects or just with the antennae of tethered insects also necessarily involves chemical cues, we separated these two modalities by extracting lipids from the antennae of *P. americana* and replacing them in some insects with a lipid extract of the body; the antennae and body contain similar cuticular hydrocarbons. Female *B. germanica* developed their oocytes at comparable rates whether they interacted with lipid-free or lipid-fortified antennae, as long as they experienced tactile stimulation. These results further confirm that chemical cues are not involved in social modulation of oocyte maturation and that the appropriate tactile stimuli alone can accelerate reproduction in *B. germanica* females.

Recent evidence suggests that group size-dependent acoustic signals from wing-fanning behavior mediate sonotactic orientation and group joining in *B. germanica* [63]. Although we did not explicitly investigate whether acoustic cues can modulate the rate of oocyte maturation, three lines of indirect evidence argue against this proposition. First, when an isolated female was separated by a screen from other females, auditory cues, if produced, failed to stimulate faster oocyte maturation. Second, in experiments where a female was tethered outside the arena, with only her antennae interacting with the test female, acoustic signaling was not possible, yet oocyte maturation was induced. On the other hand, when the antennae of the tethered female were ablated, the “grouping effect” was abolished. And third, when other insect species were paired with *B. germanica* females, it is unlikely that these insects could produce auditory cues that would be relevant to German cockroach females, yet the latter experienced a robust “grouping effect”. Nevertheless, acoustic stimuli, however unlikely, need to be more explicitly considered as potential facilitators of reproduction in the German cockroach.

Earlier studies with locusts differed in their conclusions regarding the social stimuli that induce gregarization [38], [39]; however, recent studies agree that tactile stimuli are the main sensory modality responsible for socially-induced developmental changes in nymphs [38]–[41], and similar conclusions have been reached with cockroach nymphs [8], [12], [64]. Therefore, it is not surprising that tactile stimulation is also the main mechanism through which social facilitation of reproduction occurs in adult females of the German cockroach. Our results showing that interaction with moving glass beads failed to stimulate oocyte maturation suggest that qualitative features of the tactile stimuli are important. Moreover, interactions with a freshly-killed female also failed to stimulate oocyte growth, suggesting that movement may be an important feature of the tactile stimuli. Pair-housing females with several different insect species suggested that in addition to movement, characteristics such as body size and morphology, antenna type and texture, activity period (nocturnal vs. diurnal), and behavior might affect oocyte maturation in *B. germanica*. For example, interactions with ants, which exhibit aggressive behavior, have short antennae and can emit noxious semiochemicals (e.g., formic acid) suppressed oocyte growth in cockroaches. Beetle larvae, which have rudimentary antennae and differ dramatically from cockroaches in body morphology, also failed to stimulate oocyte growth in *B. germanica*. Insects with slightly smaller or similar body size to the German cockroach had moderate effects (beetle adult, stink bug adult, fly adult, smokybrown cockroach nymph), whereas larger insects with longer antennae, namely adult cockroaches and orthopteroid species (katydid adult, brownbanded cockroach female, and camel cricket adult), had an even more pronounced effect when paired with a *B. germanica* female. These results suggest that the length, activity level, and general accessibility of the antennae during social interactions may be important in reproduction, as they appear to be in promoting nymphal development in *B. germanica* [12].

The experiment with *B. germanica* females tethered either within the experimental arena (i.e., contact with all body parts) or outside the experimental arena (i.e., contact only with the antennae) suggests that “grouping” stimuli originate mainly from antennae, but the rest of the body can also provide these stimuli. Because social interactions between intact and antennectomized females stimulate the full “grouping effect” in the intact female, it appears that contact with either the antennae or the rest of the body, independently can stimulate oocyte maturation. In support of this inference, contact only with the active antennae of *P. americana* was as effective as being paired with a conspecific female. Furthermore, contact stimulation with an artificial “antenna”—a microfibett grafted as a prosthetic “antenna” onto the antennal pedicel of *P. americana*—produced a partial grouping effect. We therefore conclude that antennal morphology and activity are integral components of the tactile stimuli, and that the female antennae are the main receiver of these social cues.

### How do females receive the mechanosensory information relevant to the “grouping effect”?

Mechanoreceptive sensilla are broadly distributed throughout the body surface of insects, with particularly high density on the legs and on sensory appendages associated with the head and cerci. In *S. gregaria* the development of gregarious behavior in nymphs is elicited through tactile input to the antennae [65], but Simpson et al. [40] also identified specialized mechanoresponsive sensilla on the outside surface of the hind femur as the primary site of tactile input [41], [66], [67]. Even in a relatively narrow clade of closely related insect species, however, morphological and reproductive phase change can be triggered by tactile stimulation of markedly different sensory organs, because in the Australian plague locust (*Chortoicetes terminifera*) the antennae are the sole organ receiving tactile stimulation [68], and the antennae appear to be the main organ involved in maternal determination of progeny phase in *S. gregaria* [25].

In addition to mechanoreceptive cells that are housed in many antennal sensilla, the cockroach antenna also possesses hair plates, campaniform sensilla and chordotonal organs that respond to tactile stimulation [50]. We attempted to determine the role of the antennae in modulating oocyte maturation in *B. germanica* using antennectomy. Indeed, females with intact antennae matured their oocytes faster than antennectomized females that were paired with them. However, this surgical manipulation proved to exert traumatic side-effects because isolated, antennectomized females developed their oocytes even slower than similarly isolated intact females, and when both paired females were antennectomized their oocyte growth over the 6 day experiment was negligible. It is possible that loss of sensory input in antennectomized females caused them to move less, have fewer social encounters with other females, and eat and drink less. Since food intake is a major regulator of the reproductive cycle [69], this surgical manipulation likely confounded the “grouping effect” with other important regulators of reproduction.

A more revealing experiment allowed solitary females to interact with other females through a wide-mesh screen using only their antennae. We confirmed for *B. germanica* adult females that the antennae alone are sufficient to receive the tactile stimuli that accelerate the reproductive cycle.

### What sensory-CNS-neuroendocrine pathways are involved in social facilitation of reproduction?

Most cockroach species are nocturnal and rest in shelters during the day [70]. *B. germanica* nymphs and adults also exhibit distinct phases of foraging and sexual activity at night and they are relatively inactive when they aggregate during the day [44], [49]. Counter-intuitively, social interactions during the scotophase stimulated oocyte maturation, whereas greater contact with conspecifics during the photophase did not. The addition of shelters did not ameliorate the effects of social isolation [34]. We suspect that the mechanisms underlying these observations relate to coupling and uncoupling of sensory inputs with the neuroendocrine system through the circadian timing system.

In *B. germanica* females, JH-III regulates the reproductive rate [30], [31]. The brain integrates a multitude of extrinsic and intrinsic stimuli, including those related to social experience, and paces the activity of the CA, dictating the rate and magnitude of all JH-dependent events [28], [33]. We recently showed that social interaction with other females elevates the rate of JH production, leading to faster oocyte development and earlier onset of sexual receptivity [34]. Interestingly, sexual receptivity, production of sex pheromone, and mating—behaviors regulated by JH—are known to be expressed in a diel manner during the scotophase, consistent with our observation that socially facilitated reproduction is also expressed only during the scotophase. Therefore, we propose two regulatory mechanisms that could account for the lack of a “grouping effect” during the photophase: (a) Input pathway: The sensory input to the brain could be uncoupled, or brain sensitivity to sensory stimuli could be diminished during the photophase, and (b) Output pathway: The circadian timing system modulates the activity of enzymes in the JH biosynthetic pathway so sensory input during the photophase fails to elevate the JH hemolymph titer. The latter idea, that CA activity in the cockroach is modulated on a circadian basis, would suggest that “grouping effects” are expressed only when the CA can respond to brain directives during the scotophase. There is no evidence thus far of diel periodicity in CA activity in the cockroach. However, this is an appealing mechanism because the long-winged flight-capable morph of the cricket *Gryllus firmus* shows a robust circadian cycle in JH titer, whereas the short-winged flightless morph does not; the brain-directed release of the neuropeptide allatostatin into the CA appears to play a major role in JH regulation [71]. The titers of some other insect hormones also cycle in a circadian manner [72], so it is plausible that JH in *B. germanica* also might be under circadian timing.

How tactile stimuli get transduced into neuronal and neuroendocrine signals that regulate CA activity and JH titer in *B. germanica* is unknown. In *S. gregaria* tactile stimulation of specialized mechanoreceptive sensilla on the hind legs causes an increase in serotonin levels in the metathoracic ganglion, which appears to mediate the process of phase transition and behavioral gregarization [41], [66], [73]. It will be particularly interesting to investigate a connection between tactile stimulation, biogenic amines and JH because the actions of allatotropins and allatostatins are known to be regulated by other neuropeptides, and biogenic amines are also known to be involved in circadian gating of behavior.
